# Clinical and Epidemiological Characteristics of Patients with Functional Stroke Mimics: A Case–Control Study from Southern Portugal

**DOI:** 10.3390/brainsci15020163

**Published:** 2025-02-07

**Authors:** Miguel Domingos, Vítor Hugo Silva, Sara Schuh, Helena Correia, Pedro Palma, João Pedroso Pedro, Bruno Vila Nova, Ana Marreiros, Ana Catarina Félix, Hipólito Nzwalo

**Affiliations:** 1 Faculty of Medicine and Biomedical Sciences, University of Algarve, 8005-139 Faro, Portugal; a81238@ualg.pt (V.H.S.); a74081@ualg.pt (S.S.); a74112@ualg.pt (H.C.); a57645@ualg.pt (B.V.N.); acfelix@ualg.pt (A.C.F.); hjnzwalo@ualg.pt (H.N.); 2 Algarve Biomedical Center Research Center, 8005-139 Faro, Portugal; 3 Stroke Unit, Algarve Local Health Unit (CHUA), 8000-386 Faro, Portugal

**Keywords:** functional stroke mimics, stroke mimics, functional neurological disorders, psychogenic stroke

## Abstract

Background: Patients with functional neurological disorder presenting as stroke mimics or functional stroke mimics (FSMs) pose significant diagnostic challenges. In the acute phase, especially when patients are present within the therapeutic window for acute reperfusion treatments, a misdiagnosis of FSM can lead to unnecessary and costly interventions. Despite its clinical importance, the literature on the risk factors for FSM is limited. This study aims to compare the clinical and epidemiological characteristics of patients with FSM to those with confirmed acute ischemic stroke (AIS). Methods: This case–control study involved temporal matching between consecutive series of patients with FSM and controls with AIS from a single tertiary university hospital in southern Portugal. Results: A total of 188 patients were included: 64 cases (FSM) and 188 controls (AIS). The rate of stroke code activation and use of ambulance between was comparable between the two groups. The group of patients with FSM was younger (53.2 years vs. 69.5 years, *p* < 0.001) and had a higher proportion of females (52.4% vs. 47.6%, *p* = 0.001). There was no difference in terms of clinical severity at presentation. The proportion of specific signs, such as transcortical aphasia (3.1% vs. 20.9%, *p* = 0.014), gait abnormalities (15.6% vs. 33.9%, *p* = 0.004), and cranial nerve abnormalities (31.2% vs. 43.5%, *p* = 0.042), was lower in the FSM group compared to the AIS group. The proportion of patients on antithrombotic therapy (90.9% vs. 9.1%, *p* = 0.007) and antihypertensive drugs (78.5%, vs. 21.5%, *p* < 0.001) prior to the event was significantly higher in the AIS group. Likewise, the prevalence of cerebrovascular risk factors such as diabetes mellitus (14.3% vs. 85.7%, *p* = 0.005), arterial hypertension (23.8% vs. 76.2%, *p* = 0.001), and smoking (43.7% vs. 56.3%, *p* = 0.005) was lower in the FSM group compared to the AIS group. No statistically significant differences were observed in cholesterol levels or the prevalence of dyslipidemia between the two groups. Psychiatric comorbidities, including generalized anxiety disorder (71.4% vs. 28.6%, *p* = 0.05) and major depressive disorder (61.9% vs. 28.1%, *p* = 0.01), were more prevalent in the FSM group. Conclusions: Patients with FSM display different clinical and epidemiological profiles, with a higher likelihood of being younger, female, having prior psychiatric conditions, and lacking traditional cerebrovascular risk factors.

## 1. Introduction

Functional Neurological Disorders (FNDs) are characterized by authentic neurological manifestations, including motor and sensory impairments. These symptoms do not result from structural abnormalities in the brain, but rather from dysfunctions in network connectivity, as implied by the term [1,2]. The disorder predominantly affects younger individuals, particularly women, although the gender gap narrows with increasing age at onset [2,3,4,5]. Known risk factors for FND include psychological trauma, chronic stress, and psychiatric comorbidities such as anxiety and depression [2]. In contrast, stroke is a neurological condition resulting from a disruption in the brain’s blood supply, with symptoms persisting for more than 24 h. It is broadly classified into ischemic (85% of cases) and hemorrhagic (15%) types [6]. Stroke is one of the leading causes of death and disability worldwide, accounting for approximately 34% of global healthcare expenditures [6,7]. The prognosis of stroke improves significantly with timely reperfusion treatment [8]. Modifiable risk factors for stroke include hypertension, diabetes, and smoking, while non-modifiable factors include advanced age and family history [9,10]. A wide range of neurological manifestations, such as gait disturbance, non-epileptic seizures, cognitive decline, tremor, speech disturbances, dizziness, and focal neurological signs, can be present in patients with FND [1,2]. The management of FND is often complex, particularly when its clinical presentation closely resembles that of acute stroke; such cases are known as functional stroke mimics (FSMs). FSMs are characterized by neurological symptoms that closely resemble those of a stroke—such as weakness, speech disturbances, or sensory deficits—but occur without any underlying cerebrovascular pathology [3,5]. FSMs account for up to 2% of all suspected stroke cases [11]. As for any FND, the diagnosis of FSM only applies when the manifestations cannot be explained by other organic causes, such as seizures or metabolic disorders [3,5,11]. In general, corresponding to the epidemiology of FND [1,2], the group of FSM patients is younger, with a greater proportion of female patients [11,12]. In addition, FSM patients are less likely to be affected by cerebrovascular risk factors [3,5,11,12,13]. Similarly to other FND patients, people with FSM often have coexisting psychiatric conditions, with anxiety disorders and major depression being the most prevalent [11,12,13,14]. AIS encompasses a wide range of clinical manifestations resulting from the obstruction of a cerebral artery [9]. When assessing a patient with symptoms suggestive of AIS, stroke physicians correlate the clinical signs with the brain’s functional and vascular anatomy to identify the affected regions [8,15]. Most patients with AIS experience hemispheric strokes, which typically present contralateral motor deficits (hemiparesis), sensory deficits (hemisensory loss), visual field defects (hemianopsia), speech or language impairments (aphasia), difficulty performing purposeful motor tasks (apraxia), or reduced awareness and responsiveness to stimulus, often on one side (neglect). In contrast, when the brainstem or cerebellum is involved (infratentorial stroke), symptoms such as vertigo, dizziness, diplopia (double vision), dysphagia, ataxia, balance disturbances, and coordination impairments are more common [8,15]. Patients with FSM, however, often present with clinical signs that are incongruent or inconsistent with a well-defined neuroanatomical location or vascular territory [11,13,14]. Given the time-sensitive nature of intravenous thrombolysis, which is indicated in the first 4.5 h of symptom onset, the risk of administering the treatment to patients who are later diagnosed with FSM or a similar condition should be accommodated [11,12]. At the group level, detailed knowledge of the characteristics of FND presenting as FSM can aid in identifying specific patterns. Unfortunately, most studies on this topic are cross-sectional studies, case series, or were not specifically designed to compare the clinical and epidemiological characteristics of FSM and AIS [11,13,14,16]. This limitation hinders our ability to draw robust conclusions about the differences between these two entities, particularly in the hyperacute setting. A more detailed and systematic investigation of these differences could guide appropriate public health strategies and clinical management. Despite its clinical significance and the high incidence reported in some studies [11,12,14], data on FSMs remain limited. Notably, there are no published studies examining the epidemiological and clinical characteristics of FSMs in several countries, including Portugal. Since geographic and ethnic differences in clinical–epidemiological characteristics of FSM are recognized [11,14], local studies are also relevant when it comes to obtaining a deeper understanding of the disorder. To address these gaps, we conducted a case–control study to analyze the clinical and epidemiological characteristics of patients presenting with stroke-like symptoms in the form of FSMs and compared them with those of patients confirmed to have experienced a stroke.

## 2. Materials and Methods

A single-center case–control study with a 1:2 ratio was conducted, using all consecutive series of patients diagnosed with FSMs, regardless of clinical severity. Only cases of FSMs that met the following criteria were included: the presence of at least one symptom of altered voluntary motor or sensory function; clinical findings that were inconsistent with recognized neurological or medical conditions; no better explanation provided by another medical or mental disorder; and clinically significant distress or impairment in social, occupational, or other important areas of functioning caused by the clinical manifestations [2]. Cases (FSMs) and controls (AIS) were identified using the electronic Institutional Stroke Unit Database (ISUD) from the inception of the database (1 January 2016) to 31 December 2018. In 2019, the registry was interrupted due to the COVID-19 pandemic. The study was conducted at the Algarve University Hospital Center, the only hospital in the Algarve region of southern Portugal offering acute reperfusion treatment for AIS [17]. All AIS patients suspected to have experienced a stroke were referred to the Algarve University Hospital Center by the Portuguese National Institute of Medical Emergencies, which coordinates the pre-hospital emergency response. The nearest hospital with similar capabilities is located two hours away.

The inclusion criteria required patients to be aged 18 years or older, exhibit least one manifestation involving altered cortical functions (e.g., aphasia; neglect, hemianopsia), motor functions (e.g., hemiparesis, dysarthria), sensory functions (e.g., hemihypesthesia), or coordination functions (e.g., ataxia, dysdiadochokinesis) functions, have symptoms lasting more than 24 h, be eligible for thrombolysis, and present as normal on a brain magnetic resonance imaging (MRI) scan. Exclusion criteria included transient neurological symptoms lasting 24 h or less, the presence of any neurological condition capable of causing stroke-like manifestations (e.g., migraine or structural brain lesions, seizures or post-seizure status), not being eligible for thrombolysis; isolated manifestations compatible with a small brainstem stroke (to prevent inclusion of false-negative cases) and inexistence of a brain MRI scan. The control group comprised patients aged 18 years or older who were admitted with confirmed acute ischemic stroke eligible for acute reperfusion treatment.

Selection of controls: The exact date and time of each FSM case were retrieved from the ISUD. Based on this information, all strokes admitted on the same day, the preceding day, or the following day were identified. From these, the two most temporally adjacent cases that met the inclusion criteria were selected as controls. This selection was made by the investigators through a review of each patient’s electronic file. Files were organized chronologically by admission.

Temporal matching: This approach minimizes potential confounding factors arising from variations in clinical practices, as both cases and controls were likely evaluated by the same physician, considering that each stroke physician is assigned specific days of the week throughout the year. To further reduce the risk of indication bias, only cases under the stroke code (≤6 h of stroke onset) were included in the analysis.

Data were extracted from the ISUD by the investigators and included sociodemographic characteristics (gender, age, and marital status); psychiatric comorbidities (generalized anxiety disorder, major depressive disorder, post-traumatic stress disorder, panic disorder, and bipolar disorder); cerebrovascular risk factors (high-density lipoprotein (HDL), low-density lipoprotein (LDL), total cholesterol, blood glucose levels, dyslipidemia, diabetes mellitus, hypertension, smoking status, and family history of cerebro-cardiovascular disease); drugs taken prior to stroke onset (antidepressants, antithrombotic medication, oral contraceptives, antihypertensive medications, and illicit drugs); and process-of-care- and hospitalization-related factors (stroke code, symptom evolution during hospitalization, and initial symptom presentation). Severity was assessed using the National Institutes of Health Stroke Scale (NIHSS), in line with the hospital protocol, which requires documenting the score for all patients with AIS.

Statistical analysis was conducted using the Statistical Package for the Social Sciences (SPSS, version 28; Chicago, IL, USA). Continuous variables were summarized as the mean and standard deviation or as the median and interquartile range (IQR), while categorical and ordinal variables were reported as absolute frequencies (n) and relative frequencies (percentages, %). We only performed preplanned comparisons that were deemed scientifically relevant a priori. Bivariate statistical analysis was performed to evaluate relationships among the study variables. The Shapiro–Wilk test was used to assess the normality of variable distributions. Based on the distribution characteristics (parametric or non-parametric), either Student’s *t*-test or the Mann–Whitney U-test were applied for continuous variables. For categorical variables, Pearson’s Chi-squared test or Fisher’s exact test were employed. Ordinal variables were consistently analyzed using the Mann–Whitney U-test, regardless of the sample distribution. Statistical significance was defined as a *p*-value of less than 0.05.

This study was conducted in accordance with the principles of the Declaration of Helsinki and received approval from the Institutional Ethics Committee (UAIF 140/2023). Informed consent was waived due to the retrospective nature of the study.

## 3. Results

**Figure 1:**
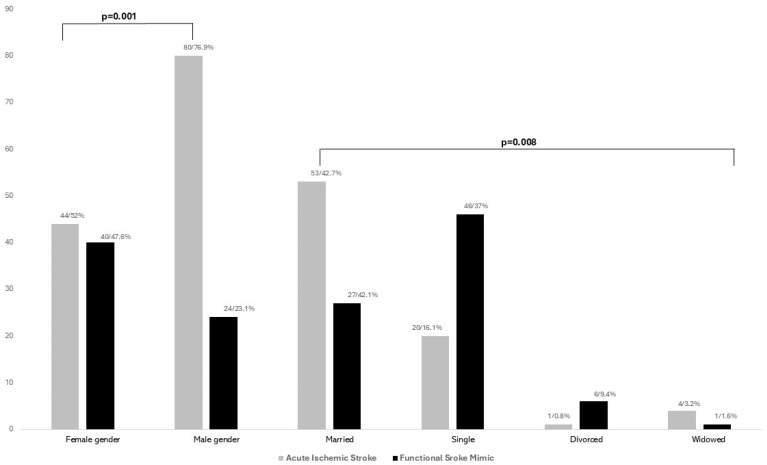
Comparison of gender and marital status distribution between patients with acute ischemic stroke and functional stroke mimics.

A total of 188 patients were included: 64 cases of FSM and 188 controls (AIS). The overall mean age was 63.9 (±16.7), with the group of FND patients being younger (53.2 vs. 69.5, <0.001), having higher proportion of females (52.4% vs. 47.6%, *p* = 0.001), and different distribution of marital status (Figure 1).

There were no statistically significant differences between FSM and AIS with regard to the frequency of pre-hospital stroke code activation (n = 19/29.6% vs. n = 36/29%, *p* = 0.263) or ambulance arrival rates (n = 3757.8% vs. n = 91/73.3%, *p* = 0.699).

There was no difference in the severity of clinical manifestations at hospital presentation, with a comparable distribution of NIHSS scores between FSM and AIS (Table 1). Among the specific clinical manifestations analyzed, significant differences were observed, with a lower proportion of transcortical aphasia (3.1% vs. 20.9%, *p* = 0.014), gait abnormalities (15.6% vs. 33.9%, *p* = 0.004), and cranial nerve abnormalities (31.2% vs. 43.5%, *p* = 0.042) in patients with FSM.

A higher proportion of headaches and vertigo was observed in patients with FSM, though this difference was not statistically significant. Most FSM patients (62/96%) exhibited at least one positive sign suggestive of functional neurological disorder (FND), with clinical incongruence (87%) and distractibility (77%) being more common. No patients with AIS displayed signs suggestive of FND. Other signs were not consistently recorded or evaluated and therefore are not included in the analysis. No statistically significant differences were found in the proportion of most other clinical manifestations.

**Figure 2:**
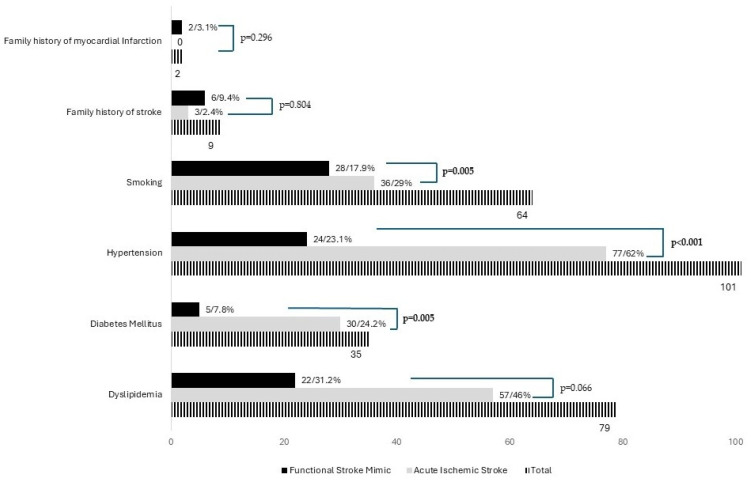
Comparison of cerebrovascular risk factors distribution between patients with acute ischemic stroke and functional stroke mimics.

The prevalence of cerebrovascular risk factors differed between FSM and AIS (Figure 2), with a higher proportion of smoking (29% vs. 17.9%, *p* = 0.005), hypertension (62% vs. 23.1%, *p* = 0.001), and diabetes mellitus (24.2% vs. 7.8%, *p* = 0.005) observed in the AIS group.

Regarding the prior history of psychiatric disorders, Table 2 shows that the prevalence of generalized anxiety disorder prior to the event was significantly higher in the FSM group compared to the AIS group (15.6% vs. 3.2%, *p* = 0.005). Similarly, the prevalence of major depressive disorder was also higher in the FSM group (20.3% vs. 6.4%, *p* = 0.01).

The proportion of patients receiving antithrombotic therapy (16.1% vs. 3.1%, *p* = 0.007) and antihypertensive drugs (58.9% vs. 31.2%, *p* < 0.001) prior to the event was significantly higher in the AIS group compared to the FSM group.

No statistically significant differences were observed in cholesterol levels or the prevalence of dyslipidemia between the two groups (Table 3).

## 4. Discussion

The clinical and epidemiological characteristics of FSM, particularly in the acute setting, have been poorly studied. This study from Portugal examines and compares the characteristics of FSM patients with those of AIS patients. Our study revealed a significant difference in average age between the groups, with the FSM group being, on average, 16.3 years younger than the stroke group. Additionally, there was a higher proportion of women within the FSM group. Similar findings were reported by Jones et al. and Wilkins et al., who observed an average age gap of 10 years and a greater proportion of women in their FSM and FND cohort [1,13,14,18,19]. The age difference may be attributed to the obvious progressive contribution of different medical comorbidities as the population gets older [9,10]. The observed gender disparity can likely be explained by several factors. Women are more likely to experience childhood adversity and are at a higher risk of anxiety, depression, and trauma. Additionally, they tend to seek medical help when facing a range of psychological or physical complaints [20,21,22].

Our study found that prior to the event, the presence of specific psychiatric disorders, particularly major depression and generalized anxiety, was common in the FSM group. The coexistence of psychiatric comorbidities, especially generalized anxiety disorder and major depressive disorder, is frequently observed in patients with both FND and FSM [1,13,14]. Various psychological factors, such as stressful life events and a history of psychological or physical abuse, may contribute to maladaptive brain functioning or neural network dysfunctions, possibly through dysregulation of the hypothalamic–pituitary–adrenal axis. These factors can affect biologically vulnerable individuals and form the basis of FND [23,24,25].

The stress hypothesis is further supported by the finding of a relatively higher proportion of windowed patients in the FSM group in our study. Recognizing and addressing these comorbidities may not only alleviate psychiatric symptoms but may also contribute to the management of patients with FSM. Consistent with previous studies [13,14,18,23], our findings demonstrated that the prevalence of cerebrovascular risk factors such as diabetes mellitus, hypertension, and a family history of stroke was lower in the FSM group. In contrast, no significant differences were observed in cholesterol levels or the incidence of dyslipidemia between the two groups. This result is likely explained by the high prevalence of dyslipidemia in our country, where nearly half of the general population has cholesterol levels exceeding recommended thresholds [26].

Apart from the underrepresentation of transcortical aphasia, gait disturbance, and cranial nerve manifestations in FSM, we found no significant differences in the clinical characteristics, including NIHSS scores, between patients with FSM and those who had experienced a stroke. Other studies have also reported that very few specific clinical manifestations can reliably differentiate FSM from AIS at the group level [13,14]. Notably, the differences between groups varied, with FSM patients showing a lower frequency of aphasia and moderate-to-severe NIHSS scores [13], or a higher frequency of left hemiparesis and facial asymmetry [14]. However, comparing these studies is challenging due to significant differences in methodology and the management of AIS and FSM. For example, variations in pre-hospital response or the organization of stroke care across regions [27] can result in patients with AIS arriving at the hospital later, and often with higher NIHSS scores compared to FSM patients.

On the other hand, the lack of standardized clinical evaluations in patients with FSM hinders the identification of more specific patterns of clinical manifestations.

To minimize the risk of bias, we restricted our study to patients arriving at the hospital under the stroke code. To improve the comparability of clinical and sociodemographic findings between studies, it is essential to establish and implement a consensus on the clinical evaluation and reporting of FSM cases.

Additionally, no differences in stroke code activation or ambulance transportation usage were observed between the groups. This finding may be explained by Portugal’s universal healthcare system, which ensures activation of the stroke code for any suspected AIS within the time window for acute reperfusion. Our study has important limitations. Patients with more pronounced or obvious clinical manifestations of FSM features—such as Hoover’s sign, marked drift without pronation, give-way weakness, or global or inverse pyramidal patterns of weakness [12]—may have had their stroke code aborted after the initial evaluation and, as a result, were not included in the database. Furthermore, data from the COVID-19 pandemic (2019–2022) were excluded due to disruptions in the stroke care chain during that period [27], which altered patterns of admissions and investigations for suspected stroke cases. This exclusion further reduced our already small sample size, diminishing the statistical power of our analysis and increasing the likelihood of failing to detect statistically significant differences or associations (Type II error). In addition, due to the retrospective nature of our study, some relevant variables such the specific professional activity, years of education were not considered due to inconsistent registration. By excluding patients with FSM and AIS who arrived outside the stroke code, we may have excluded individuals with specific clinical and epidemiological characteristics. Therefore, the findings of our study should not be generalized to patients with a clinical onset exceeding six hours.

One of the major strengths of our study is the strict inclusion of patients with extensive investigations, including brain MRI, which allowed us to exclude other potential stroke mimics. We acknowledge the possibility of false-negative cases of stroke, particularly in those with very small posterior circulation lesions. Despite the high sensitivity of MRI, particularly in hyperacute AIS, false-negative results can occur in up to 6.8% of cases [28]. However, the diagnosis of FNS was made based on a combination of positive and negative signs suggestive of FND, and the MRI scan was very rarely performed in the hyperacute phase, reducing the possibility of false-negative imaging cases of stroke.

## 5. Conclusions

Our study confirms that patients with FSM exhibit, at a group level, discernible demographic and clinical characteristics. FSM patients tend to be younger and predominantly female, with a higher prevalence of generalized anxiety disorder and major depressive disorder and a lower prevalence of cerebrovascular risk factors. Some specific clinical manifestations such as apraxia, transcortical aphasia, and gait abnormality appear less frequently in FSM patients. Studies incorporating standardized data collection protocols are needed to provide more robust and comparable evidence regarding the relationships between demographic factors, psychiatric comorbidities, cardiovascular risk factors, and FSM. Emerging biomarkers show significant promise with regard to the early identification of acute AIS [29]. Integrating these biomarkers with clinical and epidemiological findings may enhance the development of effective strategies for early diagnosis and management. At an individual level, thrombolysis should never be delayed due to suspicion of FSM. Awareness of FSM is particularly critical beyond the hyperacute phase, as it helps prevent unnecessary interventions and costs while ensuring the timely initiation of appropriate management.

## Figures and Tables

**Table 1:** Comparison of clinical manifestations between cases of functional neurological disease and stroke.

Characteristics	Total	**AIS (n = 124)**	FSM (n = 64)	*p*-Value
NIHSS Score				0.225
Minor ≤ 5	50	32 (25.8%)	18 (28.1%)	
Moderate 5–15	43	26 (20.9%)	17 (26.6%)	
Severe ≥ 16	13	10 (8%)	3 (4.7%)	
Motor or sensorial aphasia	67	48 (38.7%)	19 (29.6%)	0.294
Transcortical Aphasia	28	26 (20.9%)	2 (3.1%)	0.014
Apraxias	9	8 (6.4%)	1 (1.6%)	0.080
Vertigo	15	9 (7.2%)	6 (9.4%)	0.821
Dysarthria	62	42 (33.9%)	20 (31.2%)	0.740
Headache	38	19 (15.3%)	19 (29.7%)	0.078
Gait abnormality	52	42 (33.9%)	10 (15.6%)	0.004
Hemiparesis	183	122 (98.4%)	61 (95.3%)	0.306
Hemianopsia	31	22 (17.7%)	9 (5.7%)	0.420
Cranial nerve abnormalities	74	54 (43.5%)	20 (31.2%)	0.042
Hemihypoesthesia	139	85 (67.7%)	54 (84.4%)	0.066

AIS: acute ischemic stroke; FSM: functional stroke mimic; *p* value was calculated using the Pearson χ^2^ test or Fisher’s exact test where appropriate.

**Table 2:** Comparison of sociodemographic factors, prior history of psychiatric disease, and drug use functional stroke mimic and stroke.

Characteristics	Total (n = 188)	AIS (n = 124)	FSM (n = 64)	*p*-Value
Prior history of psychiatric disease				
Generalized Anxiety Disorder	14	4 (3.2%)	10 (15.6%)	0.005
Major Depressive Disorder	21	8 (6.4%)	13 (20.3%)	0.01
Post-traumatic stress disorder	2	2 (1.6%)	0 (0.0%)	0.28
Panic Disorder	3	2 (1.6%)	1 (1.6%)	0.91
Bipolar Disorder	1	0 (0.0%)	1 (1.6%)	0.19
Prior to event use of drugs				
Antidepressants	25	14 (11.3%)	11 (17.2%)	0.230
Previous use of antithrombotics	22	20 (16.1%)	2 (3.1%)	0.007
Oral contraceptive medication	5	1 (0.8%)	4 (6.2%)	0.025
Antihypertensive drugs	93	73 (58.9%)	20 (31.2%)	<0.001
Illicit drugs	5	3 (2.4%)	2 (3.1%)	0.817

AIS: acute ischemic stroke; FSM: functional stroke mimic; *p* value was calculated using the Pearson χ^2^ test or Fisher’s exact test where appropriate.

**Table 3:** Comparison of cholesterol and cerebrovascular risk factors between functional stroke mimic and acute ischemic stroke.

Characteristics	Total (n = 188)	AIS (n = 124)	FSM (n = 64)	*p*-Value
HDL	125	85 (68.5%)	40 (62.5%)	0.515
Normal (≥60 mg/dL)	20	13 (10.5%)	7 (10.9%)	
Moderate Risk (40–59 mg/dL)	40	30 (24.1%)	10 (15.6%)	
High Risk (Men: <40 mg/dL: Women: <50 mg/dL)	65	42 (33.9%)	23 (35.9%)	
LDL	153	102 (82.3%)	51 (79.7%)	0.969
Normal (<100 mg/dL)	62	42 (33.9%)	20 (31.2)	
Moderate Risk (100–159 mg/dL)	64	42 (33.9%)	22 (34.3%)	
High Risk (>159 mg/dL)	27	18 (14.5%)	9 (14%)	
Total Cholesterol	155	104 (67.1%)	51 (79.7%)	0.450
Normal (<200 mg/dL)	106	70 (83.9%)	36 (56.2%)	
Moderate Risk (200–239 mg/dL)	26	20 (16.1%)	6 (9.4%)	
High Risk (≥240 mg/dL)	23	14 (11.3%)	9 (14%)	
Blood Glucose (Median mg/dL, IQR) ^b^	111.0 (41.0)	114 (91.9)	102 (30.0)	0.216

AIS: acute ischemic stroke; FSM: functional stroke mimic; *p* value was calculated using the Pearson χ^2^ test or Fisher’s exact test whenever appropriate; b: *p* value was calculated using the Mann–Whitney *U* test.

## Data Availability

Upon reasonable request, we are able to share the anonymized original database. The data are not publicly available due to legal reasons.
